# Lgl1 Is Required for Olfaction and Development of Olfactory Bulb in Mice

**DOI:** 10.1371/journal.pone.0162126

**Published:** 2016-09-07

**Authors:** Zhenzu Li, Tingting Zhang, Zhuchun Lin, Congzhe Hou, Jian Zhang, Yuqin Men, Huashun Li, Jiangang Gao

**Affiliations:** 1 Institute of Developmental Biology, School of Life Science, Shandong University, Jinan, 250100, Shandong, China; 2 Jinan First People’s Hospital, Jinan, 250011, Shandong, China; 3 The Second Hospital of Shandong University, Jinan, 250000, Shandong, China; 4 SARITEX Center for Stem Cell, Engineering Translational Medicine, Shanghai East Hospital, Advanced Institute of Translational Medicine, Tongji University School of Medicine, Shanghai, 200123, China; 5 Center for Stem Cell&Nano-Medicine, Shanghai Advanced Research Institute, Chinese Academy of Sciences, Shanghai, 200123, China; 6 Shenzhen Key Laboratory for Molecular Biology of Neural Development, Shenzhen Institutes of Advanced Technology, Chinese Academy of Sciences, Shenzhen, 518000, Guangdong, China; University of Queensland, AUSTRALIA

## Abstract

Lethal giant larvae 1 (Lgl1) was initially identified as a tumor suppressor in Drosophila and functioned as a key regulator of epithelial polarity and asymmetric cell division. In this study, we generated Lgl1 conditional knockout mice mediated by Pax2-Cre, which is expressed in olfactory bulb (OB). Next, we examined the effects of Lgl1 loss in the OB. First, we determined the expression patterns of Lgl1 in the neurogenic regions of the embryonic dorsal region of the LGE (dLGE) and postnatal OB. Furthermore, the Lgl1 conditional mutants exhibited abnormal morphological characteristics of the OB. Our behavioral analysis exhibited greatly impaired olfaction in Lgl1 mutant mice. To elucidate the possible mechanisms of impaired olfaction in Lgl1 mutant mice, we investigated the development of the OB. Interestingly, reduced thickness of the MCL and decreased density of mitral cells (MCs) were observed in Lgl1 mutant mice. Additionally, we observed a dramatic loss in SP8^+^ interneurons (e.g. calretinin and GABAergic/non-dopaminergic interneurons) in the GL of the OB. Our results demonstrate that Lgl1 is required for the development of the OB and the deletion of Lgl1 results in impaired olfaction in mice.

## Introduction

The Drosophila tumor suppressor, lethal giant larvae (Lgl), is an evolutionarily conserved and widely expressed cytoskeleton protein. Lgl is essential for the establishment and maintenance of polarized epithelia, as well as cell polarity, which is associated with the asymmetric cell division of neuroblasts during fly development [1]. Furthermore, loss of Lgl results in invasive cell behavior in the Drosophila follicular epithelium during border cell migration. In mammals, there are two genes with strong homology to Drosophila Lgl (dLgl): Lgl1, which is highly expressed in the developing brain and spinal cord in mice and Lgl2, which is highly expressed in the kidney, liver and stomach [2]. In humans, overexpression of Lgl1 inhibits migration of transformed epithelial cells [3]. Lgl1 also inhibits mouse embryonic fibroblast migration and regulates the size and number of focal adhesions. Additionally, Lgl1 regulates cell polarity, membrane dynamics, and migration rates [4, 5].

Mice homozygous for the Lgl1 mutant allele [Lgl1 (-/-)], exhibit significant brain dysplasia and die within 24 h after birth [2, 6]. Lgl1 (-/-) mice also exhibit severe hydrocephalus, expansion of the striatum, dilation of the ventricles, and damage to the ventricular zone cells. However, in the Pax2-Cre line, Cre mRNA was expressed in the OB as early as E8-8.5, and its expression was restricted to the OB, midbrain, cerebellum, and kidney. Furthermore, Pax2-Cre transgenic mice efficiently deleted the Lgl1 gene with loxP-flanked sequences in these tissues [7]. In our previous study, deletion of Lgl1 resulted in abnormal cerebellar development and impairments in motor coordination, a smaller cerebellum, reduced granule precursor cell proliferation, loss of Purkinje cells, and dendritic dysplasia, when compared to their wild type counterparts [8]. However, to date, there are no detailed investigations into the role of Lgl1 in the OB.

In the mammalian olfactory system, the OB is evolutionarily conserved and highly stratified. The layers of the OB include the olfactory nerve layer(ONL); glomerular layer(GL); external plexiform layer(EPL); mitral cell layer(MCL); internal plexiform layer(IPL); and granule cell layer(GCL). The interneurons of the GCL and GL of the OB arise from the dLGE or SVZ/RMS and migrate as neuroblasts rostrally [9–11]. GL is a functional layer in the OB, representing sensory inputs from a single type of odorant receptors (OR). Odorants are detected by~1000 types of ORs expressed by olfactory sensory neurons (OSNs). Information from OSNs is relayed to second-order neurons, mitral (MCs) and tufted cells (TCs). MCs, are one of two type projection neurons in the OB that sends its axons to the olfactory cortex, and are located in the MCL. TCs, located in the EPL, project a single primary dendrite into a single glomerulus to receive synaptic inputs from the axons of olfactory sensory neurons and send their axons to the olfactory cortex. MCs also project a single primary dendrite into a single glomerulus with TCs, making reciprocal synapses with the dendrites of PG cells [12]. Odor signals, which are processed within the glomerulus, propagate to the EPL, along the primary dendrites of MCs and TCs. The signals subsequently arrive at the cell body of TCs located in the EPL and MCs in the MCL. In this study, we conditionally deleted the Lgl1 gene in the OB by crossing Lgl1 (Flox/Flox) mice with Pax2-Cre [7]. The result of the decrease of interneurons in the GL, alteration of MCL thickness and the notable decrease of MCs all likely suggest their importance for odor signal propagation, thereby resulting in impaired olfaction.

## Materials and Methods

### Ethics Statement

All animal experimental procedures were approved by Ethics Committee of Shandong University. Animal management was performed strictly in accordance with the standards of the Animal Ethics Committee of Shandong University (Permit Number: ECAESDUSM 20123004).

### Animals

The following mouse lines were used and genotyped: Lgl1 (Flox/ Flox) (MGI accession number: 102682) (Olga Klezovitch, Seattle, USA), Pax2-Cre (MGI accession number: 3046196) (Andrew K. Groves, Los Angeles, CA) [2,7]. All lines were of a hybrid C57BL/6J and 129/Sv background. Mice were housed in sound-attenuated rooms under constant temperature (22°C), lights on from 4:00 a.m. to 4:00 pm, and access to water and food ad libitum. Once vaginal plugs were detected, this day was considered to be embryonic day 0.5 (E0.5). The day of birth was defined as postnatal day 0 (P0). The embryos for experiments were harvested at E12.5 and E14.5 respectively. Prior to perfusion, mice were given a lethal dose of Euthatal anaesthetic by hypodermic injection (0.7%, 1.5μl/kg). The expression of Pax2 Cre-recombinase is restricted to OB, stria vascularis, cerebellum, midbrain and kidney, but no pallium where the source of OB interneurons and striatal projection neurons from during the construction of OB [7, 13].

We genotyped wild-type mice (WT), heterozygous mice and homozygous mice (HOMO) by PCR. DNA was extracted from mice tail snips for PCR analysis. The PCR genotyping of the floxed Lgl1 allele was detected by the following primers: Lgl1-L (5′-TACTGTGATCAGCCCAAGACTG) and Lgl1-R (5′- GGAGGATCCCAAGATAGAGGAC). Mice were genotyped for Cre using the following primers: Cre-F (5′-AGCTAAACATGCTTCATCGTCGGTC-3′) and Cre-R (5′-TATCCAGGTTACGGATATAGTTCATG-3′).

### Olfaction behavioral testing

Two independent behavioral tests for olfaction were performed. An observer was blinded to genotype information during the trials: buried food pellet and olfaction maze test [14, 15]. The buried food pellet test eliminated the need for pretraining to detect an exogenous food odor. Mice were placed on a food-restricted diet (0.2 g chow/mouse/24h) daily for 3 days before testing and during the 4 test days. Mice were allowed free access to water. Before each trial, mice were habituated to the experimental setting by placing in the test cage (45-24-20 cm). Each trial was conducted daily for 3 testing days. In each trial, one mouse was placed in the test cage to recover a 0.5 g food pellet buried 0.5 cm below the surface of a 3 cm deep layer of mouse bedding materials. The location of the pellet was changed at random before every test. The latency time was recorded and defined as the time between placement of the mouse in the cage and grasping the food pellet with its forepaws or teeth. Mice were allowed to consume the pellet and then returned to their cage. The bedding in the test chamber was changed between two trials. As a control, a visible pellet test was performed identically, and the food pellet was placed randomly on top of the bedding materials. This control test was carried out on all mice after each daily trial, and mice were allowed to consume the food pellet.

In the “olfaction maze test,” a maze bounded by a circular wall with a 76.2-cm diameter was constructed. The maze had six open corners, evenly spaced within a circular enclosure. The corners obscured the food pellet from sight such that the pellet could only be detected by smell. Mice were placed on a food-restricted diet, as described in the“buried food pellet”test, before testing and during the 3-day experimental period. Before the first trial, the mice were familiarized with the maze for 10 min daily over 3 days. For the experimental trial, a mouse was placed at the center of the maze, and a 0.5 g food pellet was placed behind one of the corners at random. The latency was defined as the time between when the mouse was placed in the maze and grasped the food pellet. The mouse was allowed to consume the food pellet before returning to the cage. Before every trial, the maze was cleaned with 70% ethanol and water to remove dirt and odorants. As a control, after each experimental trial, mice were exposed to a visible food pellet placed at the perimeter of the circular enclosure but no corners.

The data in the figures and legends were given as mean ± standard error of the mean (SEM). Parameters were compared with a two-tail paired Student *t*-test. All data represent mean± SEM (*P<0.05, **P<0.01). The value of *n* referring to the number of analyzed cells or animals for each group.

### Histology and immunohistochemistry

Wild-type and Lgl1 mutant mice were sacrificed by cervical dislocation and their cochleae were dissected from the heads of mice. To generate paraffin sections, mouse tissues or embryos were fixed in 4% paraformaldehyde at 4°C overnight and then dehydrated via an ethanol series. The tissues were embedded with the proper orientation in paraffin, sectioned at 10-μm thickness and stained using hematoxylin and eosin (H&E) for microscopic analysis.

For frozen sections, tissues were fixed in 4% paraformaldehyde at 4°C for 3–4 h and infiltrated with 30% sucrose (in PBS) at 4°C overnight. Tissues were embedded in OCT compound and frozen using isopentane cooled by liquid nitrogen. The tissue blocks were stored at -40°C and sectioned at 10 μm.

For immunostaining, an immunofluorescence assay was performed using standard staining procedures with the following primary antibodies and secondery antibodies: polyclonal rabbit anti-Hugl-1 (Santa Cruz, Heidelberg, Germany, 1:50); anti-SP8 (Santa Cruz, Heidelberg, Germany, 1:50); monoclonal mouse anti-Nestin (Chemicon, Temecula, CA, 1:200); monoclonal mouse anti-Tbx21(Abcam, Shanghai, China, 1:50); Alexa Fluor 593/488-labelled goat anti-mouse (Invitrogen, Carlsbad, CA, 1:300) and Alexa Fluor 593/488-labelled goat anti-rabbit IgG (Invitrogen, Carlsbad, CA, 1:300).

## Results

### Lgl1 expression in the dLGE and postnatal OB

**Fig 1:**
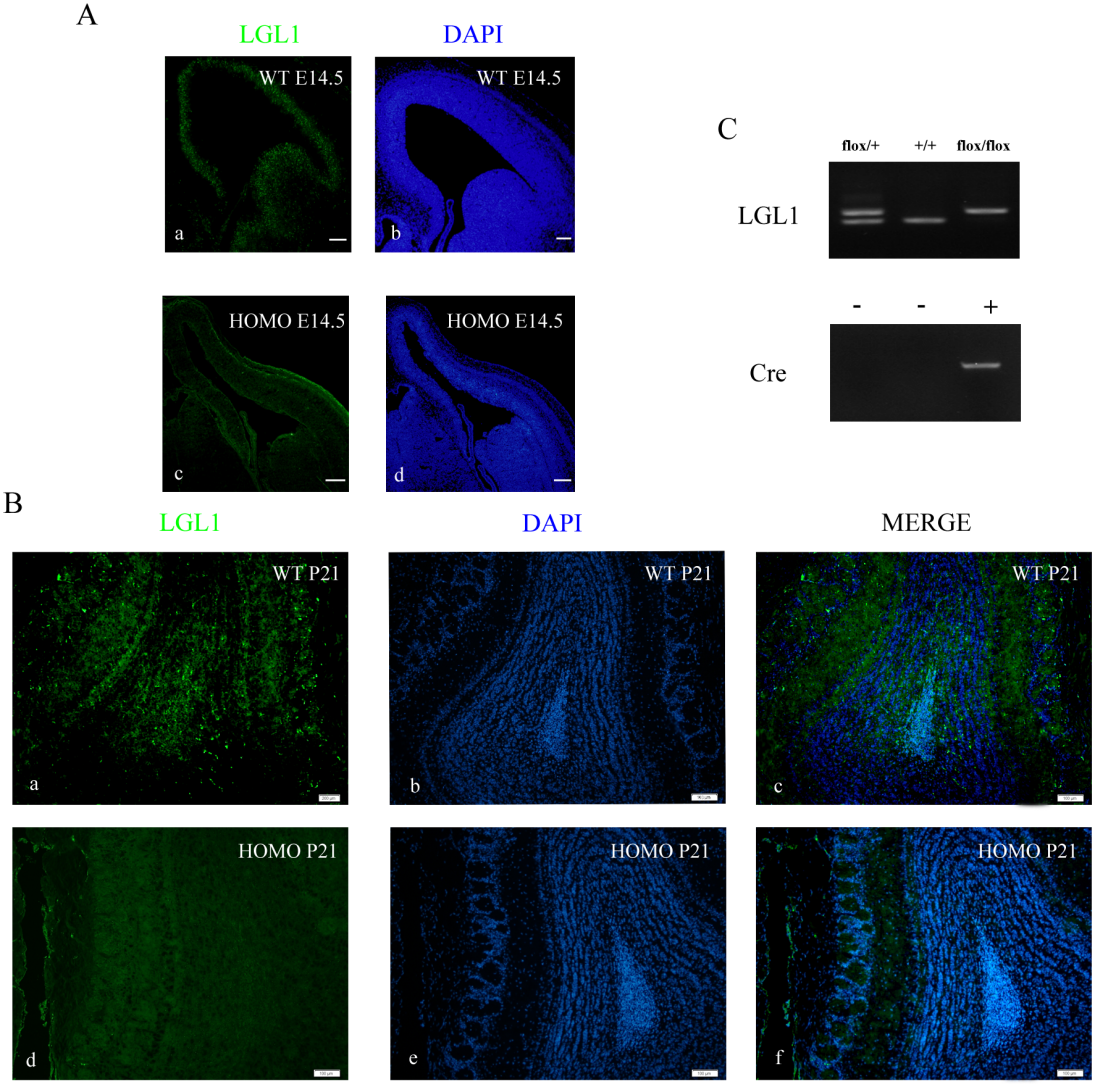
Generation of Lgl1 conditional knockout mice mediated by Pax2 Cre mice. Immunofluorescence staining was performed on frozen sections of E14.5 and P21 wildtype and mutant dLGE using an anti-Lgl1 antibody. Nuclear marker DAPI was used for counter staining (blue). (A) Lgl1 was highly expressed in dLGE of wildtype mice and efficiently deleted in mutant mice. (B) Lgl1 was highly expressed in OB of wildtype mice and efficiently deleted in mutant mice. (C) Genotyping of Lgl1-Pax2 Cre cko mice via PCR. Lanes: heterozygous (Flox/+), homozygous (Flox/Flox) and wildtype (+/+) mice. Scale bars represent 50 μm.

We generated Lgl1 conditional mutants by mating mice with Lgl1 flox/flox and a Pax2 Cre mice in which Cre-recombinase is expressed in the OB [7]. To determine the expression and effective deletion of Lgl1 in the dLGE and OB, we performed immunofluorescence using an antibody that specifically recognized amino acids 935–1036 at the C-terminus of Lgl1 on SVZ and OB sections from wildtype mice at E14.5 and postnatal stages P21 respectively. We found that Lgl1 protein was detected throughout the dLGE and OB and highly expressed in the MCL, GCL and GL in WT. As expected, Lgl1 was efficiently deleted in the LGE (Fig 1A) and OB of mutant mice (Fig 1B and 1C).

### Conditional inactivation of Lgl1 results defects in the OB

**Fig 2:**
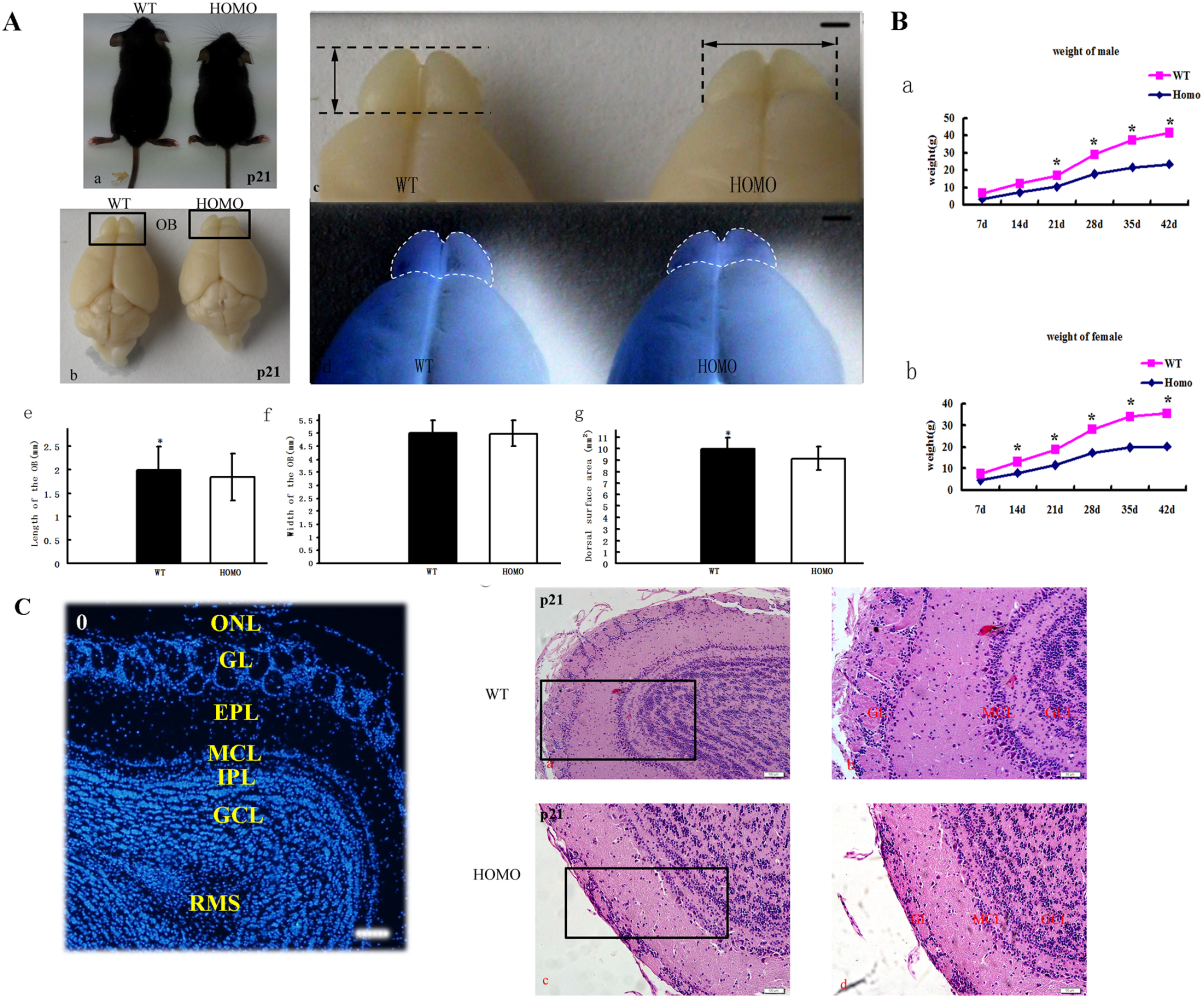
The defects of OB in Lgl1 mutants. (A) The difference in body (a) and OB (b) size between wildtype and mutant two-week-old male littermates. Lgl1 conditional mutants developed a relatively normal looking brain with noticeably smaller olfactory bulbs. Length and width were measured using a vernier caliper as indicated by arrows in c, and the dorsal surface area was quantified as indicated by dotted lines in d. (e, g) The mean length and surface area of OB were significantly different between WT and Lgl1 mutants, but no obviously changes of width (means±se, *P<0.05, paired *t* test, n = 9 mice/group). Scale bars: 1 mm (c, d). (B) A decrease in body weight between the wild and mutant animals was observed both in female and male mice. The significant difference of weight showed at p21 in males, while at p14 in females between wild and mutant animals (means±se, *P<0.05, paired *t* test, n = 9 mice/group). (C). (0) The laminar structure of OB. (a-d) A notable decrease in density and disorganization of granule cells at p21. The boundaries of the GL and GCL were fuzzy or lost in the OB of Lgl1 conditional mutants. Sections stained by H&E. (b) Boxed regions in (a), (d) and (c). ONL, olfactory nerve layer; GL, glomerular layer; EPL, external plexiform layer; MCL, mitral cell layer; IPL, internal plexiform layer; GCL, granule cell layer, RMS, rostral migratory stream. Scale bars represent 1mm in Ac and 200 μm(0), 100 μm (a,c) and 50 μm (b,d) in C.

First, we compared the weight and size of OB of WT and Lgl1 mutants (Fig 2). As shown in Fig 2Ab, we measured both the length and width of the OB using a vernier caliper after the dorsal part of the skull was removed immediately, avoiding the artifacts caused by removal. Also we measured the dorsal surface area of the OB after according to the previous report [16]. As shown in Fig 2e and 2g, the mean length and dorsal surface areas of Lgl1 mutants OB were significantly smaller than WT (means±se, *P<0.05, paired *t* test, n = 9 mice/group), however the width between Lgl1 mutants and WT was not different from each other (width, P = 0.47). There was also a significant decrease in average weight of litter size in both females and males at the stages examined.

As described previously, the OB is a conservative laminated structure (Fig 2C0). Nneurons and their axonal connections are organized into distinct layers, which correspond to different functionalities in odor processing. The GL was severely damaged and disrupted in the OB of Lgl1 mutants. By P21, when the mature cytoarchitecture has been established in control mice, the OB appeared disorganized in Lgl1 mutants, especially the flatted GL and MCL (Fig 2Ca–2Cd). In the mutants, we observed no obvious distinctions between the laminar structures. We also noted disorganization of the MCL. Moreover, in the GL, the glomeruli appeared to only be stacked on top. Indeed, in most cases, the density appeared to be decreased in mutants, possibly due to the reduced size of the OB. Conditional inactivation of Lgl1 in the dLGE and OB of mutant mice resulted in a size reduction of the OB, as well as an altered cytoarchitecture. Additionally, we observed a decrease in body weight of both female and male mutant mice compared to their WT counterparts (Fig 2B, *P<0.05, paired *t* -test, n = 9mice/group).

### Impaired olfaction in Lgl1 mutant mice

**Fig 3:**
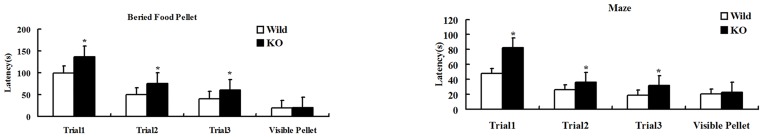
Impaired olfaction in Lgl1 mutant mice revealed by behavioral testing. Olfactory performance was tested in buried food pellet and olfactory maze tests as described above. A) Buried food pellet test. The latency for mice to recover a buried food pellet was recorded on each of 3 consecutive testing days (means±se, *P<0.05, paired *t* test n = 9 mice/group). Latency times are not significantly different in a control visible pellet test (P = 0.16). B) Olfactory maze test. The latency for mice to recover a hidden food pellet was recorded (means±se, *P<0.05, paired t test, n = 9 mice/group;). Latency times are not significantly different in the control visible pellet test (P = 0.14).

Olfactory behavioral test measures the general olfactory ability of mice. The olfactory ability has been divided into primary or secondary hierarchical organized levels, which include identification, discrimination or odor recognition and ratings. The ability of identification is to perceive and name an odor or something, while discrimination is to differentiate between/among a set of odors. For example, a simple task is to recognize whether odors exit and to determine the location. In this study, behavioral analysis of olfaction was performed using the buried food pellet recovery test and the olfaction maze test. In the buried food pellet test, latency to find the buried food pellet is measured for food-deprived mice [14, 15]. As indicated in Fig 3A, there is a significantly greater latency for Lgl1 mutants on each trial day (means±se, *P<0.05, paired *t* test, n = 9 mice/group). For the control condition, there were no differences in latency to recover visible the food pellet between the WT and Lgl1 mice (P = 0.17).

As previously described, we performed an olfactory maze test to confirm impaired olfaction in Lgl1 mutants. As shown in Fig 3B, conditional latency times were significantly greater for the Lgl1 mutants versus the WT mice (means±se, *P<0.05, paired *t* test, n = 9 mice/group), but no significant difference in latency times was detected in the control test condition (P = 0.15). These observations indicated that olfaction was impaired in Lgl1 mutants.

### Inactivation of Lgl1 results defective in dLGE

**Fig 4:**
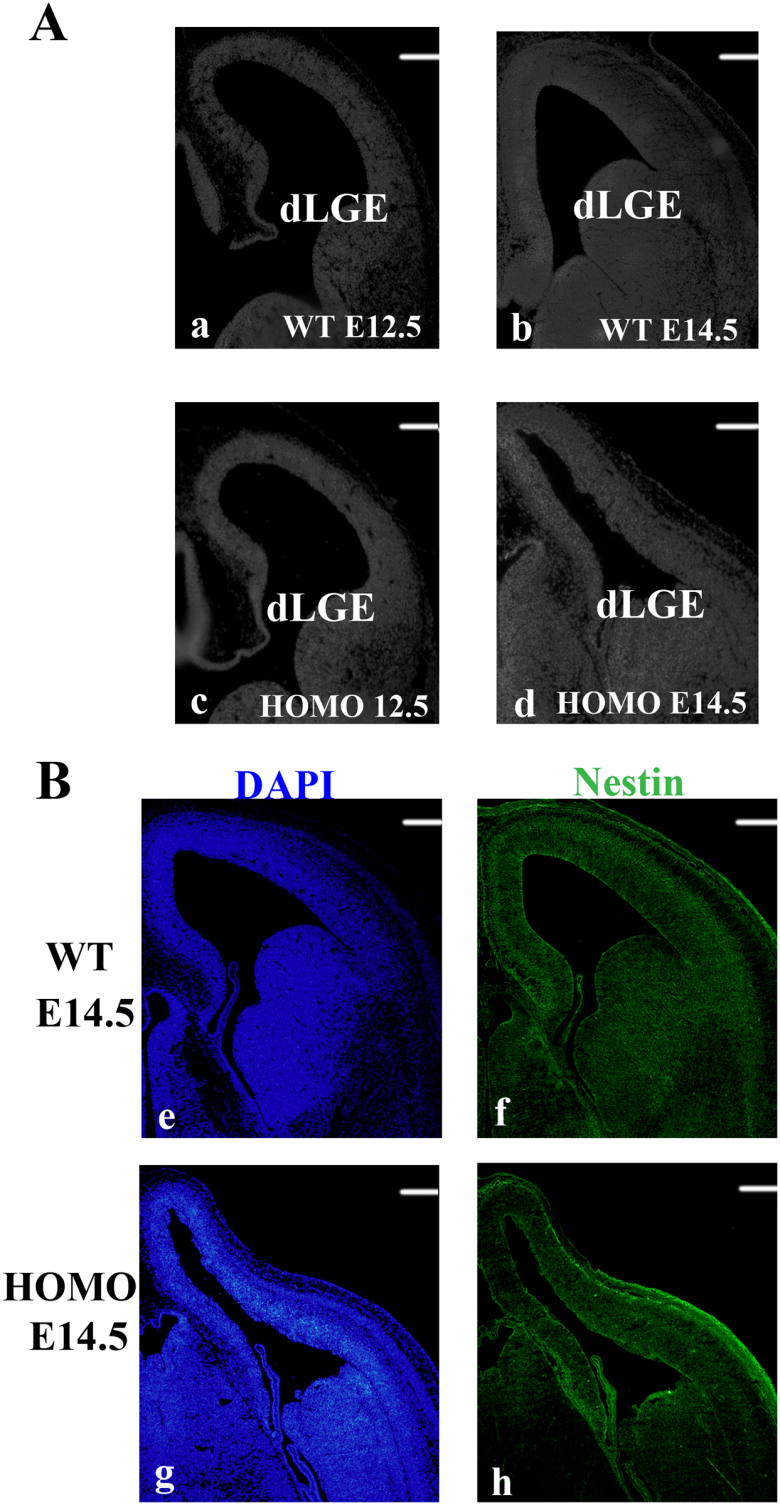
The defects of dLGE in the Lgl1 mutants. (A) At E12.5, there were subtle changes in progenitor zone of the dLGE, while an apparent collapse in morphology at E14.5. (B) Expression of Nestin in the dLGE. Progenitor cell staining by Nestin appeared normal at E14.5. This indicated that progenitor cells of interneurons in the OB could arise from the dLGE normally. dLGE, dorsal lateral ganglionic eminence. Scale bars represent 50 μm.

To investigate the cellular mechanisms responsible for impaired olfaction, we assessed constructional changes of the OB. Puelles and his co-workers have proposed that the pallium consists of four major subdivisions extending from the dorsocaudal to ventrorostral regions: mid, dorsal, lateral and ventral pallium (MP, DP, LP and VP). The LP consists of the olfactory cortex [17]. The transient embryonic structure, LGE, where most of the striatum and pallidum developed from, is the source of OB interneurons and striatal projection neurons. Furthermore only cells from the LGE are able to migrate efficiently from the adult SVZ to OB [18]. A previous study has suggested that, at embryonic stages, OB interneurons arise from the progenitor domain in the dLGE [18]. To determine the effect of Lgl1 on the formation of the dLGE, we investigated the molecular properties of the dLGE at earlier stages in Lgl1 mutant mice. Lgl1 mutant mice displayed a smaller dLGE at E14.5, while developing a dLGE-like construction. However, at E12.5, there were subtle changes in progenitor zone of the dLGE (Fig 4Aa and 4Ab). By E14.5, the defects in the Lgl1 mutants were more apparent. The dLGE was smaller compared to WT littermates (Fig 4Ac and 4Ad). Additionally, expression of Nestin, a marker for central nervous system progenitor cells, appeared normal at E14.5in the dLGE (Fig 4B). The results indicated that although there were defects in the dLGE, interneurons of the OB could still arise from the progenitor domain normally.

### Reduced thickness of the MCL in Lgl1 mutants with a decrease in density of mitral cells

**Fig 5:**
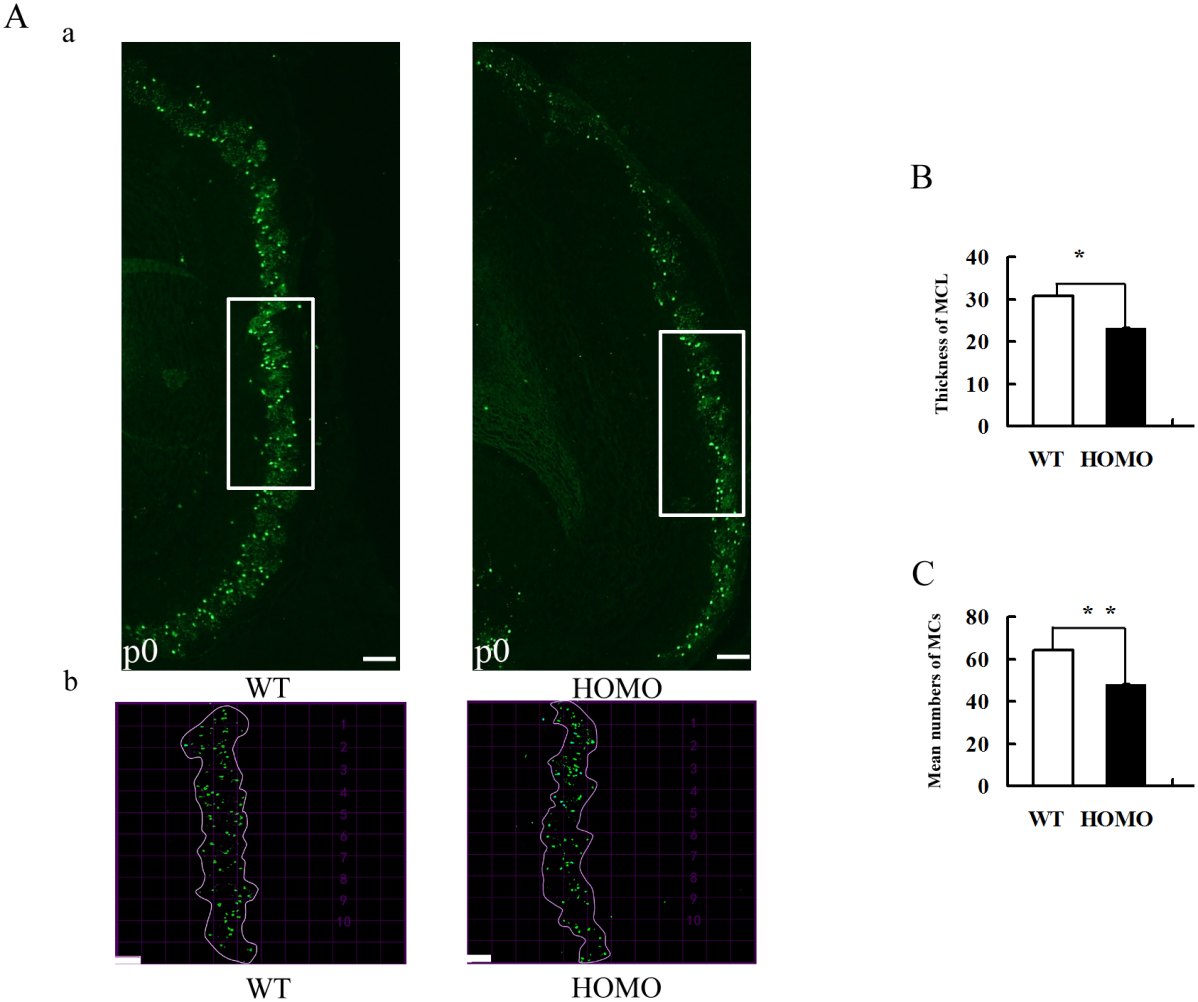
Morphometric analysis of MCL thickness and number of MCs in WT and mutants at P0. (A) Representative micrographs showing Tbx21-positive MCs in the OB (green). Appropriate grids (20×20μm) were applied, and the average thickness of the MCL was calculated from the measurement at 10 grid lines that marked, (b) Boxed region in (a). (B) Bar graph indicating that the thickness of the MCL in Lgl1 mutants was significantly decreased compared with that of WT mice at P0 (means±se, *P<0.05, **P<0.01, paired *t* test, 9 mice/group;). (C) The graph shows that the mean number of MCs in Lgl1 mutants was significantly different from that of WT mice (means±se, *P<0.05, **P<0.01, paired *t* test, 9 mice/group;). Scale bars represent 200 μm (A, a), 20 μm(A, b).

In odor information process, MCs of MCL receive signal inputs from OSNs at glomeruli, and then send output to cortical areas. To confirm the changes in MCL thickness and the number of MCs in mutants, we performed immunofluorescence on MCs, stained with an antibody against Tbx21, a molecular marker for MCs nucleus in the OB. As indicated in Fig 5A and 5B, the Tbx21-positive layer was significantly thinner in Lgl1 mutants compared to WT mice at P0 (means±se, *P<0.05, paired *t* test n = 9 mice/group).

Previous reports have shown the involvement of Lgl1 in the regulation of migration and hyperproliferation [2, 5]. Thus, we also examined the number of MCs using antibody Tbx21. The results indicate the mean number of MCs in the MCL was significantly higher in WT versus Lgl1 mutant mice. (Fig 5C, means±se, *P<0.05, **P<0.01, paired *t* test, n = 9 mice/group). These results indicated that Lgl1 might play a crucial role in maintaining the normal levels of MCs by impacting the total number of cells.

### Disorientation of SP8^+^ interneurons in GL

Sp8, a new member of the Sp1 zinc-finger transcription factor gene family [19, 20], can be used to identify specific populations of OB interneurons. Notably, calretinin and GABAergic/non-dopaminergic populations persist in differentiating OB interneurons of the GCL and GL. Calretinin is an intracellular calcium-binding protein that belongs to the troponin C superfamily and plays a role in many cellular functions. It was concluded that in the interneuron populations, calretinin and GABAergic/non-dopaminergic interneurons are most dependent on Sp8 function for their normal development and survival [20]. In this research, we mainly investigated the changes in whole populations of sub-interneurons in the GL. To determine whether there were consequences of Lgl1 deletion on SP8^+^ interneuron formation of GL, immunofluorescence was performed using an antibody against SP8 to identify these interneurons.

**Fig 6:**
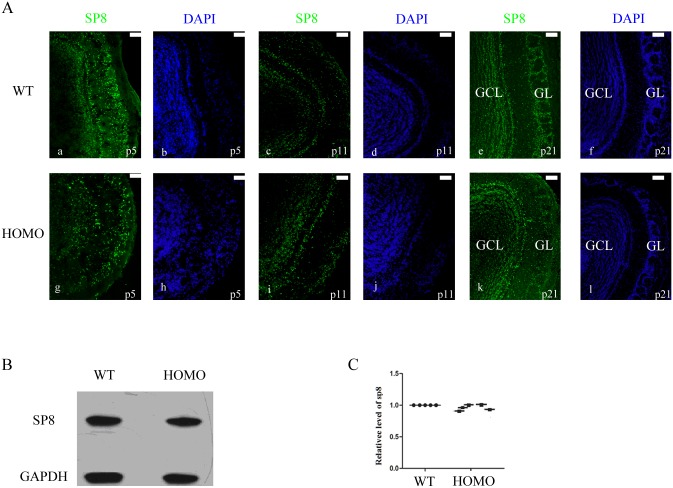
The disorganized nature of SP8^+^ interneurons in GL and the expression level of SP8^+^ interneurons. (A) Lgl1 conditional mutants exhibited defects in the OB interneurons distribution. Lgl1 conditional mice exhibited severe disorganization in OB interneurons and an almost complete loss of interneurons in the GL at P21. There was a noticeable decrease of SP8^+^ interneurons in the GL of Lgl1 mutants (f^,^) compared to WT controls (f). (B) Western blotting analysis of the total protein extracted from the OB of wild type and Lgl1 mutants at P21 revealed no decrease in the expression of SP8 in Lgl1 mutant mice compared to the WT mice (means±se, *P<0.05, **P<0.01, paired *t* test, 9 mice/group). Scale bars represent 50 μm.

At the stages examined, SP8^+^ interneurons were not reduced in the GL of Lgl1 conditional mutants compared to controls at the stages P5 and P11(Fig 6A, 6B and 6D). However, at P21, there was an obvious decrease in SP8+ interneurons of the GL in OB in the mutants.

Neuroblasts, derived from SVZ, migrated to the OB via the rostral migratory stream (RMS). Once they reached the OB, they began radial migration and matured into interneurons. The migration emerged from the SVZ, coalesced into the RMS, and ended in the OB, persisting in life [21, 22]. The disorganized location of SP8^+^ interneurons in the mutant GL suggests that there was abnormal migration and orientation of these interneurons, an effect could contribute to impaired olfaction. Next, western blotting analysis was performed to analyze the expression of SP8^+^ at P21 (Fig 6B). These results also revealed no changes in the expression levels of SP8 in the mutant mice compared to WT mice. These results indicate that Lgl1 may play a crucial role in organizing and maintaining the normal distribution and orientation of interneurons in the GL, however Lgl1 may not impact SP8 protein levels in the interneurons of the GL.

## Discussion

In this study, we generated Lgl1 conditional knockout mice in OB by mating mice with Lgl1 flox/flox and a Pax2 Cre mice and reported that the mutation of Lgl1 in mice results in severe inhibition of OB development and impaired olfaction. Indeed, abnormal morphology and impaired olfaction is considered one of the most significant features in some diseases.

### Lgl1 is required for the development of dLGE and OB

Previous studies indicated that More-Cre-mediated Lgl1 knockout mice exhibited significant brain dysplasia and died within 24 h after birth. Using a conditional knockout strategy, we have uncovered a crucial requirement for Lgl1 in the regulation of OB development and olfaction. Moreover, Lgl1 may play a significant role in organizing and maintaining the normal cytoarchitecture of the OB, especially in maintaining typical laminar structure. Lgl1 mutants have a smaller dLGE at E14.5, and develop a dLGE-like construction. Given the noticeable reduction in the size of the OB, it is possible that the disruption of dLGE results in a considerable reduction in size. Also, the Lgl1 conditional mutants exhibit abnormal laminar structure compared to that of the OB in WT mice mutants also do not exhibit a distinct IPL, EPL or MCL. These results suggest that Lgl1 may play a crucial role in organizing and maintaining the normal cytoarchitecture of OB. Interestingly, similar alterations in cytoarchitecture were observed in Arx and Prokr2 mutants, which also exhibited severe reductions in OB interneurons [23, 24]. Future studies investigating whether Lgl1 is intact embryonically and conditionally inactivated at postnatal stages may help to address this previous observation.

### Lgl1 deficiency results in impaired olfaction by affecting development of the OB

Lgl1 mutant mice performed poorly on olfactory-based behavioral tasks as compared to WT mice, suggesting that Lgl1 deletion leads to deficits in olfactory-mediated behavior. Although the mechanisms through which the Lgl1-induced deficiency leads to olfactory dysfunction in mice is unknown, our observations suggest that Lgl1 may play a vital role in olfaction. Immunofluorescence analysis revealed that Lgl1 is localized in GL and GCL layer cells of the OB. In addition to the OB, Lgl1 is also expressed in other brain regions, such as the hippocampus and cerebellum, which are important for higher-level odor tasks and motor coordination. In our previous study using Lgl1 conditional knockout mice, the mutants exhibited defects in the cerebellum, resulting in impairments of motor coordination [8], but there was no evidence suggesting that the motor coordination was a major influencer on olfaction. The impairments in olfaction and motor coordination may explain the reductions in body weight observed in this study.

**Fig 7:**
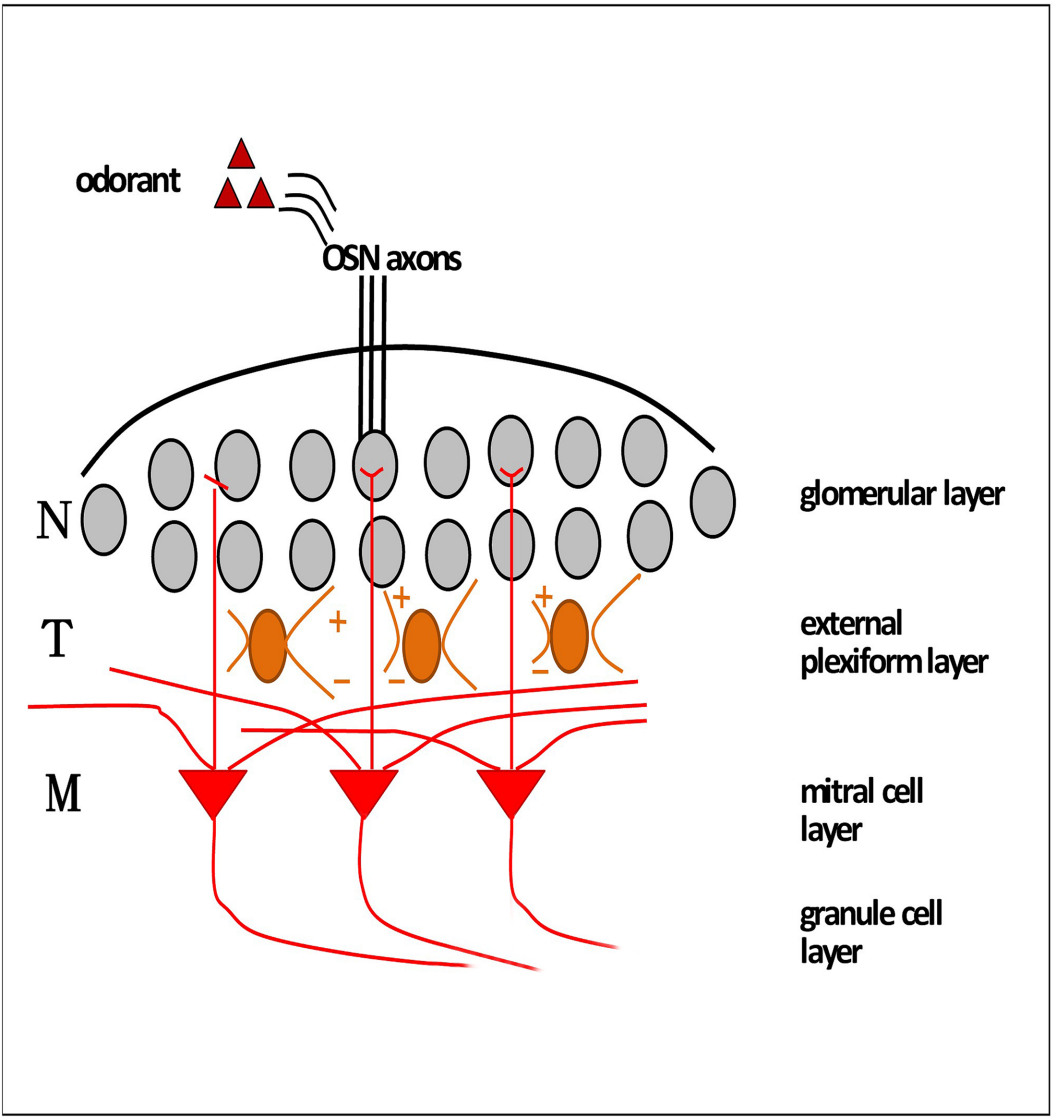
The roles of interneurons in GL and MCs in odor information process. Each glomerulus receives convergent axonal inputs from OSNs expressed by a given OR, where odorants are detected. Within a glomerulus, interneurons function as the odor information relay station, where the second neurons, MCs/TCs tufts, ramify within. The mitral/tufted cell typically possesses a single primary dendrite and several lateral dendrites. MCs receive signal inputs from OSNs at glomeruli, and send output to cortical areas. Granule cells and deep short axon cells receive centrifugal feedback from the olfactory cortex. TCs receive excitatory input from MCs and then send inhibitory feedback. +, receive excitatory; -, inhibitory feedback. N, interneuron; T, tufted cell; M, mitral cell.

Within a glomerulus, interneurons function as the odor information relay station, and the loss of SP8^+^ interneurons may lead to interruptions in odor information processing. A typical glomerulus is innervated by primary dendrites of 20 to 50 MCs/TCs, among which, 5 to 20 are MCs [25, 26]. It is known that there are at least three distinct populations of interneurons in the GL: the ones that express calbindin, calretinin, or a synthetic enzyme for GABA production, which are classified by the expression of the calcium-binding proteins and glutamic acid decarboxylase [27, 28]. Although the functional distinctions of calbindin and calretinin remain elusive, SP8 is expressed in most migrating neuroblasts and remains in subpopulations of mature OB interneurons [29]. In this study, there is a decrease in SP8^+^ interneurons in the GL. The disorganized SP8^+^ interneurons in the GL of mutant mice might suggest abnormal migration and orientation of GL interneurons, which could result in defects of olfaction. The loss of SP8^+^ interneurons in the GL might suggest the responsibility for the impaired olfaction (Fig 7).

Neuroblasts normally migrate through the RMS using chain migration [18], when reach the OB, they switch to radial migration in order to reach their final position in the GCL and GL. The conditional Lgl1 mutant’s neuroblasts appeared to be severely impaired in the dLGE. A striking effect observed in Lgl1 mutants was a marked reduction in size of the OB. In addition, the typical laminar structure of the OB was abnormal. In fact, the GL, MCL, and IPL were difficult to distinguish anatomically. In addition to disruptive morphology, there was also a significant loss of SP8^+^ interneurons in the GL and reduced MCs in the MCL of Lgl1 mutant mice. Although the mechanism of regulation in the distribution and expression of interneurons of the OB is not clear, the abnormal distribution and orientation of neurons in the GL and MCL of Lgl1 mutants suggest that these changes might result in impaired olfaction.

### The role of Lgl1 in human diseases

Lgl1 was initially found to be a tumor suppressor protein in flies and a key regulator of epithelial polarity and asymmetric cell division [30]. In Drosophila, Lgl acts together with Ras to promote aggressive cancers, though no direct evidence shows Lgl1 participates in carcinogenesis in higher eukaryotes. A previous study indicated that the loss of Lgl1 in mammals results in loss of cell polarity and subsequent loss of asymmetric cell divisions of neural progenitor cells. These changes cause defects in the cell cycle and differentiation, which is characteristic of primitive neuroectodermal tumors [5]. In undifferentiated glioblastoma cells—which are glioblastoma tumor initiating cells—the inactivation of Lgl1, helps to maintain glioblastoma tumor initiating cells in the undifferentiated state. Besides the role of Lgl1 in tumorigenesis, loss of Lgl1 also leads to a loss of the Fragile X Mental Retardation Protein, which controls the architecture of NMJ [31]. Lgl1 could interact with Rab10 GTPase directly in the polarized delivery of membrane-precursor vesicles to distal axonal projections in mammals [32]. Based on these results, we hypothesized that Lgl1 might be involved in the morphogenesis and neurogenesis of OB. As mentioned previously, the Lgl1 mutant mice exhibited a smaller OB size with almost complete loss of interneurons in the GL, reduced projection neurons in the MCL and impaired olfaction. Though the structural abnormalities of olfactory epithelium (OE) and the central cortices are considered to be early features of Alzheimer and Parkinson's diseases, it was concluded that the initial establishment of the OB central projections is able to proceed independently of the olfactory sensory axons from the OE, and there was no evidence demonstrated the relationship between OE and OB in mammals directly [33]. In this study no notably abnormal morphology of OE detected. Olfactory impairments are common non-motor features, which occurs at least 90% of cases. In this study, using the strategy of conditional knock out, we found that the loss of Lgl1 led to abnormal morphological characteristics, impaired olfaction and defects in the development of OB neurons. The mutant mice genenreted by Lgl1-Pax2 provided a model to study the function of Lgl1, both in olfaction and related human disease.

## References

[1] Assemat E, Bazellieres E, Pallesi-Pocachard E, Le Bivic A, Massey-Harroche D. Polarity complex proteins. Biochim Biophys Acta. 2008; 1778: 614–30. . 18005931 10.1016/j.bbamem.2007.08.029

[2] Klezovitch O, Fernandez TE, Tapscott SJ, Vasioukhin V. Loss of cell polarity causes severe brain dysplasia in Lgl1 knockout mice. Genes Dev.2004; 18:559–571. . 15037549 10.1101/gad.1178004 PMC374237

[3] Schimanski CC, Schmitz G, Kashyap A, Bosserhoff AK, Bataille F, Schäfer SC, et al Reduced expression of Hugl-1, the human homologue of Drosophila tumour suppressor gene Lgl1, contributes to progression of colorectal cancer. Oncogene. 2005; 24: 3100–9. 10.1038/sj.onc.1208520 . 15735678

[4] Plant PJ, Fawcett JP, Lin DC, Holdorf AD, Binns K, Kulkarni S, et al A polarity complex of mPar-6 and atypical PKC binds, phosphorylates and regulates mammalian Lgl1. Nat Cell Biol. 2003; 5: 301–8. 10.1038/ncb948 . 12629547

[5] Dahan I, Yearim A, Touboul Y, Ravid S. The tumor suppressor Lgl1 regulates NMII-A cellular distribution and focal adhesion morphology to optimize cell migration. Mol Biol Cell.2012; 23: 591–601. 10.1091/mbc.E11-01-0015 . 22219375 PMC3279388

[6] Tallquist MD, Soriano P. Epiblast-restricted Cre expression in MORE mice: a tool to distinguish embryonic vs. extra-embryonic gene function. Genesis. 2000; 26: 113–115. . 10686601 10.1002/(sici)1526-968x(200002)26:2<113::aid-gene3>3.0.co;2-2

[7] Ohyama T, Groves AK. Generation of Pax2-Cre mice by modification of a Pax2 bacterial artificial chromosome. Genesis. 2004; 38: 195–199. . 15083520 10.1002/gene.20017

[8] Congzhe H, Lingcui D, Jian Z, Yecheng J, Chen S, Zhenzu L, et al Abnormal cerebellar development and Purkinje cell defects in Lgl1-Pax2 conditional knockout mice. Developmental Biology. 2014; 395: 167–181. 10.1016/j.ydbio.2014.07.007 . 25050931

[9] Luskin MB. Restricted proliferation and migration of post-natally generated neurons derived from the forebrain subventricular zone. Neuron.1993; 11: 173–189. . 8338665 10.1016/0896-6273(93)90281-u

[10] Lois C, Alvarez-Buylla A. Long-distance neuronal migration in the adult mammalian brain. Science. 1994; 264: 1145–1148. . 8178174 10.1126/science.8178174

[11] Pencea V, Luskin MB. Prenatal development of the rodent rostral migratory stream. J. Comp. Neurol. 2003; 463:402–418. 10.1002/cne.10746 . 12836176

[12] Nagayama S, Hommaand R, Imamura F. Neuronal organization of olfactory bulb circuits. Frontiers in Neural Circuits. 2014; Volume 8, Article 9. 10.3389/fncir.2014.00098 . 25232305 PMC4153298

[13] Edwards DA, Thompson ML, Burge KG. Olfactory bulb removal vs peripherally induced anosmia: differential effects on the aggressive behavior of male mice. Behav Biol. 1972; 7: 823–828. 4676291 10.1016/s0091-6773(72)80174-3

[14] Nathan BP, Yost J, Litherland MT, Struble RG, Switzer PV. Olfactory function in apoE knockout mice. Behav. Brain Res. 2004; 150: 1–7. 10.1016/S0166-4328(03)00219-5 . 15033273

[15] Schmouth JF, Banks KG, Mathelier A, et al Retina restored and brain abnormalities ameliorated by single-copy knock-in of human NR2E1 in null mice. Mol Cell Biol. 2012; 32: 1296–1311. 10.1128/MCB.06016-11 . 22290436 PMC3302440

[16] Schablitzky S, Pause BM. Sadness might isolate you in a non-smelling world: olfactory perception and depression. Frontiers in Psychology. 2014; Volume 5, Article 45. . 24570666 10.3389/fpsyg.2014.00045 PMC3916769

[17] Stenman J, Toresson H, Campbell K. Identification of two distinct progenitor populations in the lateral ganglionic eminence: implications for striatal and olfactory bulb neurogenesis. J. Neurosci. 2003a; 23: 167–174. . 12514213 10.1523/JNEUROSCI.23-01-00167.2003 PMC6742158

[18] Wichterle H, Garcia-Verdugo JM, Herrera DG, Alvarez-Buylla A. Young neurons from medial ganglionic eminence disperse in adult and embryonic brain. Nat. Neurosci. 1999; 2: 461–466. 10.1038/8131 . 10321251

[19] Yun K, Potter S, Rubenstein JL. Gsh2 and Pax6 play complementary roles in dorsoventral patterning of the mammalian telencephalon. Development. 2001; 128: 193–205. . 11124115 10.1242/dev.128.2.193

[20] Bell SM, Schreiner CM, Waclaw RR, Campbell K, Potter SS, Scott WJ. Sp8 is crucial for limb outgrowth and neuropore closure. Proc. Natl. Acad. Sci. 2003; 100: 12195–12200. 10.1073/pnas.2134310100 . 14526104 PMC218735

[21] Capilla-Gonzale V, Cebrian-Silla A, Guerrero-Cazares H, Garcia-Verdugo JM, Quiñones-Hinojosa A. The generation of oligodendroglial cells is preserved in the rostral migratory stream during aging. Frontiers in Cellular Neuroscience. 2013; Volume 7, Article 147. 10.3389/fncel.2013.00147 . 24062640 PMC3775451

[22] Wichterle H, Garcia-Verdugo JM, Alvarez-Buylla A. Direct evidence for homotypic, glia-independent neuronal migration. Neuron. 2014; 18: 779–791. . 9182802 10.1016/s0896-6273(00)80317-7

[23] Yoshihara S, Omichi K, Yanazawa M, Kitamura K, Yoshihara Y. Arx homeobox gene is essential for development of mouse olfactory system. Development. 2005; 132: 751–762. . 15677725 10.1242/dev.01619

[24] Prosser HM, Bradley A, Caldwell MA. Olfactory bulb hypoplasia in Prokr2 null mice stems from defective neuronal progenitor migration and differentiation. European Journal of Neuroscience.2007; Vol. 26: 3339–3344. 10.1111/j.1460-9568.2007.05958.x . 18052978 PMC2228368

[25] Sosulski DL, Bloom ML, Cutforth T, Axel R, Datta SR. Distinct representations of olfactory information in different cortical centres. Nature. 2011; 472: 213–6. 10.1038/nature09868 . 21451525 PMC3354569

[26] Ke MT, Fujimoto S, Imai T. A simple and morphology-preservingoptical clearing agent for neuronal circuit reconstruction. Nat Neurosci.2013; 16: 1154–61. 10.1038/nn.3447 . 23792946

[27] Kosaka T, Hataguchi Y, Hama K, Nagatsu I, Wu JY. Coexistence of immunoreactivities for glutamate decarboxylase and tyrosine hydroxylase in some neurons in the periglomerular region of the rat main olfactory bulb: possible coexistence of gamma-amino-butyric acid (GABA) and dopamine. Brain Res. 1985; 343: 166–171. . 2864104 10.1016/0006-8993(85)91172-2

[28] Kosaka K, Toida K, Aika Y, Kosaka T. How simple is the organization of the olfactory glomerulus? The heterogeneity of so-called periglomerular cells. Neurosci. Res. 1998; 30: 101–110. . 9579643 10.1016/s0168-0102(98)00002-9

[29] Kosaka T, Kosaka K. Further characterization of the juxtaglomerular neurons in the mouse main olfactory bulb by transcription factors, Sp8 and Tbx21. Neuroscience Research. 2012; 73: 24–31. 10.1016/j.neures.2012.02.013 . 22387948

[30] Lee CY, Robinson KJ, Doe CQ. Lgl1, Pins and aPKC regulate neuroblast self-renewal versus differentiation. Nature. 2006; 439: 594–598. 10.1038/nature04299 . 16357871

[31] Zarnescu DC, Jin P, Betschinger J, Nakamoto M, Wang Y, Dockendorff TC, et al Fragile X protein functions with lgl and the par complex in flies and mice. Dev. Cell. 2005; 8: 43–52. 10.1016/j.devcel.2004.10.020 . 15621528

[32] Wang T, Liu Y, Xu XH, Deng CY, Wu KY, Zhu J, et al Lgl1 activation of rab10 promotes axonal membrane trafficking underlying neuronal polarization. Dev. Cell. 2011a; 21: 431–444. 10.1016/j.devcel.2011.07.007 . 21856246

[33] Lopez-Mascaraque L, de Castro F. The olfactory bulb as an independent developmental domain. Cell Death and Differentiation. 2002; 9: 1279–1286. 10.1038/sj.cdd.4401076 . 12478464

