# Correction: Lgl1 Is Required for Olfaction and Development of Olfactory Bulb in Mice

**DOI:** 10.1371/journal.pone.0182505

**Published:** 2017-07-27

**Authors:** Zhenzu Li, Tingting Zhang, Zhuchun Lin, Congzhe Hou, Jian Zhang, Yuqin Men, Huashun Li, Jiangang Gao

The following information is missing from the Funding section: This work was supported by a grant from the Natural Science Foundation of Shandong Province (grant No. ZR2015PC020).
